# Enhanced or Weakened Western North Pacific Subtropical High under Global Warming?

**DOI:** 10.1038/srep16771

**Published:** 2015-11-26

**Authors:** Chao He, Tianjun Zhou, Ailan Lin, Bo Wu, Dejun Gu, Chunhui Li, Bin Zheng

**Affiliations:** 1 Institute of Tropical and Marine Meteorology (ITMM), China Meteorological Administration (CMA), Guangzhou, China; 2 LASG, Institute of Atmospheric Physics (IAP), Chinese Academy of Sciences (CAS), Beijing, China; 3 Joint Center for Global Change Studies (JCGCS), Beijing, China

## Abstract

The Western North Pacific Subtropical High (WNPSH) regulates East Asian climate in summer. Anomalous WNPSH causes floods, droughts and heat waves in China, Japan and Korea. The potential change of the WNPSH under global warming is concerned by Asian people, but whether the WNPSH would be enhanced or weakened remains inconclusive. Based on the multi-model climate change projection from the 5th phase of Coupled Model Intercomparison Project (CMIP5), we show evidences that the WNPSH tends to weaken and retreat eastward in the mid-troposphere in response to global warming, accompanied by an eastward expansion of East Asian rain belt along the northwestern flank of WNPSH. Weakened meridional temperature gradient on the northern flank of WNPSH and the associated thermal wind account for the weakened WNPSH in the mid troposphere. We recommend the WNPSH be measured by eddy geopotential height (*He*) instead of traditionally used geopotential height, especially in climate change studies.

In summer, the subtropical western North Pacific (WNP) is controlled by a high pressure system associated with anticyclone circulation in the mid and lower troposphere. This high pressure system is called Western North Pacific Subtropical High (WNPSH), also named Bonin High or Ogasawara High by Korean and Japanese scientists^1^,^2^. The WNPSH transports water vapor into East Asia via southerly wind on its western flank, and anchors the rain belt on its northwestern periphery where moist southerly wind meets the cold air mass^3^,^4^. The excessive or deficient rainfall along the Meiyu-Baiu-Changma rain belt in China, Korea and Japan is regulated by WNPSH^2^,^3^,^4^,^5^. Meanwhile, the areas near the ridgeline of the WNPSH are dominated by sunny hot days due to descending motion^6^. Anomalous WNPSH causes flood in some areas but heat waves and droughts in other areas of East Asia^6^,^7^, such as the great flood along the Yangtze River in 1998^3^,^8^, the drought from central China to Korean and Japan in 1994^9^, and the record-breaking heat wave over Japan in 2010^6^. Tropical cyclone activities over WNP are also regulated by the WNPSH^10^,^11^.

The decadal change of the WNPSH in the late 1970s has caused a regime shift of East Asian climate^12^,^13^,^14^, which is driven by tropical ocean warming associated with the phase reversal of Pacific Decadal Oscillation^12^,^15^. Given the significance of WNPSH to East Asian climate, how the ongoing global warming would change the WNPSH has drawn great attention. But projection studies on future climate based on model outputs are inconclusive. Some studies claimed an intensification and westward extension of the WNPSH^16^,^17^, while others argued a generally unchanged WNPSH^18^. With the output of 31 CMIP5 models^19^, we investigate the projected change of summer WNPSH using multiple metrics to obtain more robust signal in this study, by comparing the second half of 21st century in Representative Concentration Pathway 8.5 (RCP8.5) run with the second half of 20th century in Historical run.

## Results

**Figure 1:**
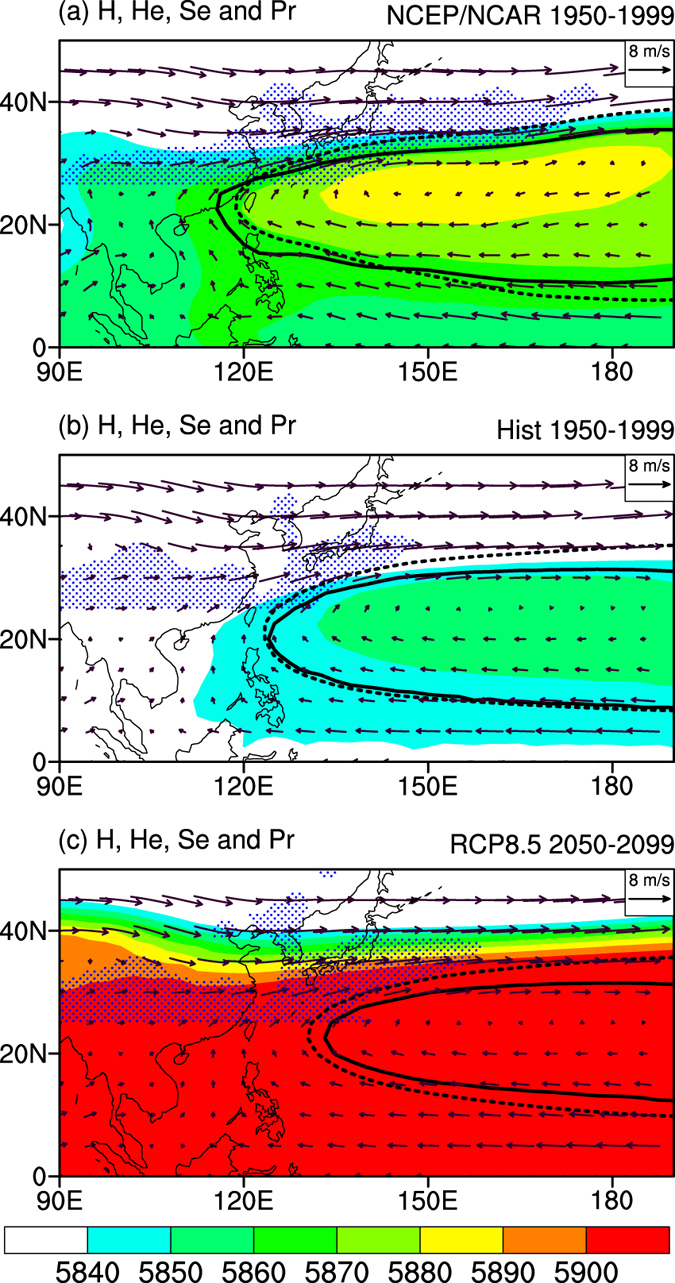
The seasonal mean state of WNPSH for June-July-August at 500 hPa. (**a**) The observation. (**b**) Historical run. (**c**) RCP8.5 run. The shading is geopotential height (“*H*”, unit: m), and the vectors are winds (unit:m s^−1^). The solid line is the 0 m contour of eddy geopotential height (*He* = 0 m), and the dashed line is the 0 m^2^ s^−1^ contour of eddy stream function (*Se* = 0 m^2^ s^−1^). The regions with a precipitation rate above 5 mm·day^−1^ north of 25˚N are filled with blue dots. This plot was created by NCAR Command Language^36^.

The WNPSH is usually measured by geopotential height (*H*) over the WNP. Higher (lower) *H* over WNP is regarded as a stronger (weaker) WNPSH. The 5880 m contour of *H* at 500 hPa is widely used as a benchmark to measure the WNPSH by meteorologists from China, Korea and Japan^1^,^17^,^20^ to determine the location of heavy rains, because East Asian rain belt is usually located on the northwest of this contour^1^,^5^,^17^ (Fig. 1a).

How does the *H* at 500 hPa change as climate warms? Compared with the Historical run (Fig. 1b), the multi-model ensemble mean (MME) for the RCP8.5 run projects a substantial increase of *H* in the late half of 21st century (Fig. 1c). The *H* values over the entire WNP south of 30˚N are below 5860 m in Historical run but are above 5900 m in RCP8.5 run. It seems that the WNPSH is intensified in RCP8.5 run according to the increase of *H* and the expansion of the area with an *H* value above 5880 m^16^,^17^. Despite the northwestward shift of *H* = 5880 m contour in a warmer climate, northwestward movement is not seen in the rain belt over East Asia, which covers Japan, Korea and southern part of China (blue dotted regions in Fig. 1b,c). The rain belt in East Asia doesn’t follow the *H* = 5880 m contour as climate warms, although this contour is a good indicator for East Asian rain belt under current climate^5^,^20^.

The nature of the WNPSH is planetary wave^21^,^22^,^23^, which is characterized by higher *H* than its surrounding regions. Here we define eddy geopotential height (*He*) as the deviation of *H* from the regional average over 0˚–40N˚, 180˚W–180˚E, following the definition in previous studies^12^,^24^. We measure the boundary of WNPSH using the *He* = 0 m contour, instead of the *H* = 5880 m contour. Since some previous studies also measure the subtropical highs using stream function related variables, we also validate our results based on the eddy stream function (*Se*) defined as the deviation of stream function from the regional average of 0˚–40˚N, 180˚W-180˚E.

In Historical run (Fig. 1b), the MME well captures the spatial pattern of the *He* = 0 m and the *Se* = 0 m^2^ s^−1^ contours, and the anticyclone wind field is also well captured. But the *H* values is systematically underestimated and the *H* = 5880 m contour cannot be found in Historical run, as noted by previous study^17^. Since the circulation is determined by the gradient of *H* rather than the absolute magnitude of *H* according to the momentum equation of atmosphere, the systematic underestimation of *H* does not affect the simulation of WPNSH. This explains why the simulated *He*, *Se* and wind field resembles the observation but the *H* does not.

**Figure 2:**
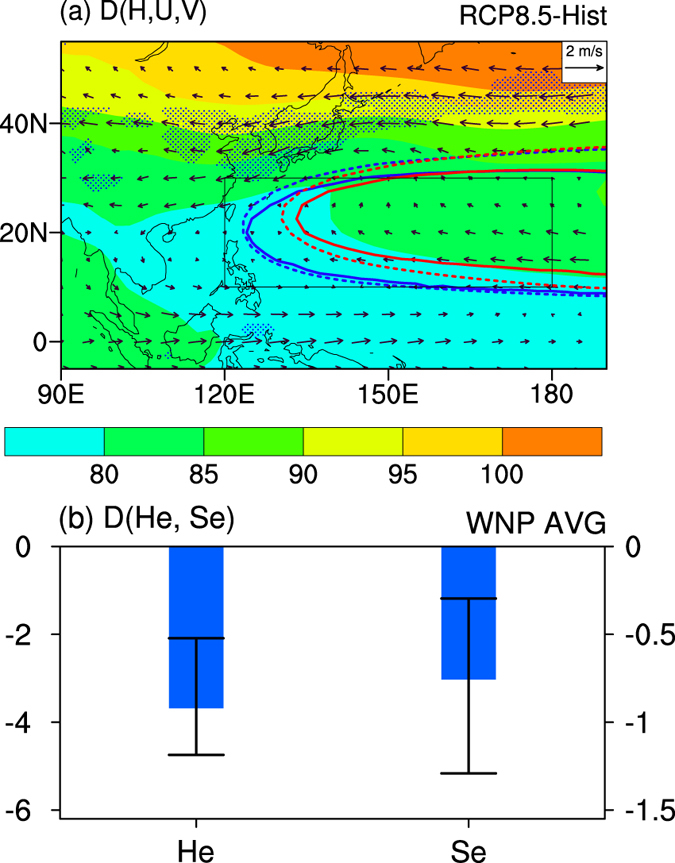
Projected changes for the WNPSH under RCP8.5 (2050–2099 average) relative to Historical run (1950–1999 average). (**a**) MME projected change in *H* (shading, unit: m) and wind (vectors, unit: m·s^−1^). The changes in wind exceeding 75% consensus among the 31 models are filled by blue dots. The solid line is the *He* = 0 m contour and the dashed line is the *Se* = 0 m^2^ s^−1^ contour. The blue and red lines stand for Historical and RCP8.5 runs, respectively. (**b**) The WNP (10˚–30˚N, 120˚E-180˚, i.e. the box in Fig. 2a) averaged changes in *He* (unit: m, left *y*-axis) and *Se* (unit: m^2^ s^−1^, right *y*-axis). The thick blue bars indicate the MME, and the thin black error bars show the range of the 25th and 75th percentiles of individual models. This plot was created by NCAR Command Language^36^.

Compared with Historical run, the *He* = 0 m and the *Se* = 0 m^2^ s^−1^ contours both contract in RCP8.5 run, and their western edge retreats eastward (contours in Fig. 2a). The contraction of the *He* = 0 m contour indicates that the increase of *H* over the WNP is smaller than the zonal mean (See supplementary Fig. S1). Indeed, decreases of *He* and *Se* over WNP (10˚–30˚N, 120˚E-180˚ averaged) are seen in more than 75% of the individual models. The MME projected decrease of *He* is 3.7 m, and the MME projected decrease of *Se* is 3.0 × 10^6^ m^2^ s^−1^ (Fig. 2b). Following the contraction and eastward retreat of the *He* = 0 m and *Se* = 0 m^2^ s^−1^ contours, the rain belt around Japan expands eastward (Fig. 1b,c). The changes in *He*, *Se* and rainfall both suggest a weakened and eastward retreated WNPSH at 500 hPa as climate warms.

The projected change of wind also indicates a weakened WNPSH. There is an anomalous cyclone wind anomaly around the *He* = 0 m (and *Se* = 0 m^2^ s^−1^) contour of the Historical run (vectors in Fig. 2a), which is primarily contributed by the weakened westerly wind on the northern flank of WNPSH. On the northern flank of WNPSH, the weakened westerly wind is agreed by more than 75% of the individual models. On the southern flank of WNPSH, weak easterly wind anomaly is seen at about 15°N but westerly wind anomaly is evident south of 10°N (vectors in Fig. 2a), both of which are below 75% of inter-model consensus. The regional average over 0°–20°N, 120°E-180° suggests a slightly weakened easterly trade wind. Our results agrees with previous studies that global warming weakens the Pacific easterly trade wind^25^,^26^, and that the reduction in the western Pacific trade wind is less robust than that in the eastern Pacific^26^.

The change of *H* is consistent with the wind changes. The increase of *H* at 500 hPa is agreed by more than 75% of the models at each grid point over WNP. The magnitude of increase is at least 70 m for MME (shading in Fig. 2a). The increase of *H* is fairly uniform on the southern flank of WNPSH but with strong meridional gradient on its northern flank. Higher increase in *H* is seen at higher latitudes (shading in Fig. 2a), and it weakens the meridional gradient of *H* on the northern flank of WNPSH. Consistent with the changes in *H* gradient, the westerly wind on the northern flank of WNPSH is substantially weakened, but the easterly trade wind on the southern flank of WNPSH is only slightly weakened.

The projected changes of *H*, *He*, *Se*, wind and precipitation consistently show a weakened and eastward retreated WNPSH. Previous study showed that the reduced intensity of WNPSH is accompanied by eastward retreat of its western boundary at intraseasonal and interannual time scales^2^,^5^, and our results confirmed this law at the centennial time scale. The results based on RCP8.5 run are also confirmed by RCP4.5 run (Supplementary Fig. S3). Previous studies suggested an enhanced WNPSH under global warming based on the rise of *H*^16^,^17^, but we argue that the weakened meridional gradient of *H* on the northern flank of WNPSH clearly indicates a weakened WNPSH.

**Figure 3:**
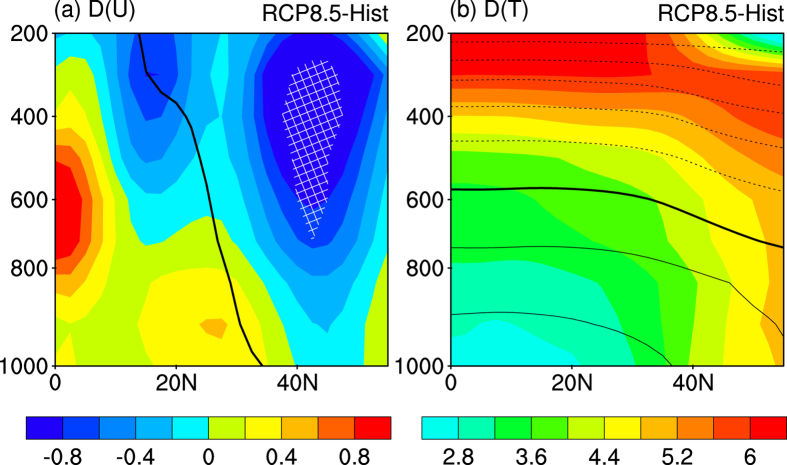
The latitude-height profile of MME projected changes averaged within 120˚–180˚E. **(a)** The changes in the zonal wind (shading, unit: m s^−1^). The black line indicates the climatological interface between trade easterly wind and mid-latitude westerly wind in Historical run. **(b)** The climatology (contours) and MME projected changes (shading) in temperature (unit: °C). The thick contour shows the 0 °C isotherm, and the contour interval is 10 °C with the negative contours dashed. The MME projected change agreed by over 75% of the individual models are filled with white crosses. The increase of temperature in (**b**) has exceeded 75% inter-model consensus everywhere, and the white-crosses are omitted. This plot was created by NCAR Command Language^36^.

What are the changes in the vertical structure of the WNPSH? As shown in the latitude-height profile of zonal wind changes (Fig. 3a), the westerly wind on the northern flank of WNPSH and the easterly wind on the southern flank of WNPSH both weakens at the mid and lower troposphere. The decelerated westerly wind on the northern flank of WNPSH is evidenced by more than 75% of the models in the mid to upper troposphere, but the inter-model consensus is low at the lower troposphere. The WNPSH is projected to be weakened in the mid troposphere but remains unchanged in the lower troposphere, consistent with the previous study which claimed weak change of WNPSH at 850 hPa^18^. The projected change of WNPSH shown here doesn’t contradict with previous studies which claimed an intensification of the subtropical high over eastern Pacific^27^, since the subtropical high over western Pacific differs from that in eastern Pacific^28^.

Why does the WNPSH weaken in the mid troposphere? As shown in the vertical structure of the projected temperature change over WNP, the warming pattern is much different for the southern and northern flanks of WNPSH (Fig. 3b). On the southern flank of WNPSH (south of 30˚N), the increase in temperature is horizontally uniform. The horizontal uniform warming in the tropics is caused by equatorial waves which efficiently smooth out the horizontal temperature gradient^29^. In contrast, the warming on the northern flank of WNPSH is characterized by strong meridional gradient (Fig. 3b), The stronger warming at higher latitudes weakens the mean state temperature gradient^30^,^31^, and therefore weakens the westerly wind in the mid troposphere according to the thermal wind law. Since the weakened WNPSH is mainly contributed by the weakened westerly wind on its northern flank, the weakened meridional temperature gradient is responsible for the weakened WNPSH.

**Figure 4:**
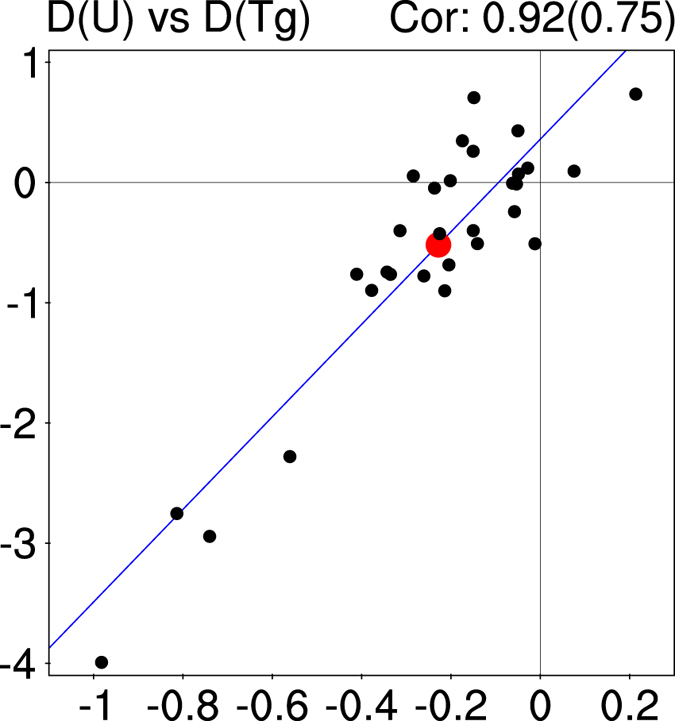
Scatter diagram for the projected changes in westerly wind as a function of meridional temperature gradient for individual models. The abscissa is the changes in the 925–500 hPa averaged meridional temperature gradient on the northern flank of WNPSH (15˚–25˚N, 120˚–180˚E minus 40˚–50˚N, 120˚–180˚E, unit: ˚C), and the ordinate is the changes in the 500 hPa westerly wind on the northern flank of WNPSH (25˚–40˚N, 120˚–180˚E averaged, unit: m s^−1^). The red dot represents the MME, and the black dots represent the 31 individual models. The inter-model correlation coefficient for the 31 models are marked on the upper-right corner, and the correlation coefficient after excluding the four outliers at the lower-left corner of the plot is marked within the parenthesis. This plot was created by NCAR Command Language^36^.

An inter-model comparison confirms the close relationship between the westerly wind and the meridional temperature gradient (Fig. 4). The larger the reduced meridional temperature gradient on the northern flank of WNPSH, the larger the weakened westerly wind. The inter-model correlation coefficient is 0.92 for the 31 models and it is 0.75 if the four outliers at the lower-left corner of Fig. 4 are removed, exceeding the 99% confidence level based on Student’s *t* test. The close inter-model connection between the westerly wind on the north of WPNSH and the meridional temperature gradient also holds under RCP4.5 (Supplementary Fig. S5). Previous studies have addressed the dominance of land-sea thermal contrast on low level subtropical highs over eastern ocean basins under global warming^27^,^32^. Our results shown above suggest that the meridional temperature gradient is the dominant factor for the WNPSH in the mid troposphere.

## Discussion

Why did many of the previous studies claim enhanced WNPSH as climate warms? It originates from the methods used in measuring the WNPSH. Traditional metrics on the WNPSH based on *H* mislead people under the warming climate^13^,^24^,^33^. According to the integrated form of hydrostatic equation, the *H* for a given pressure level *p* is proportional to the column integrated temperature (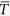) between level *p* and surface *Ps*, i.e., 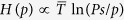. Since the global averaged *Ps* should not change due to air mass conservation, *H*(*p*) should be systematically increasing due to increasing 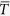
under the warming climate. This theoretical formulation is confirmed by model projections (Supplementary Fig. S1). The systematic increase of *H* may mislead people to conclude an intensification and westward extension of the WNPSH, which is inconsistent with the changes in circulation and rainfall. In fact, the increase of *H* over WNP is smaller than the zonal mean increase (Supplementary Fig. S1). It is not surprising that previous studies which claimed an intensification of WPNSH are all based on the increase of *H*^14^,^16^,^17^, without comparing the rise of *H* with changes in circulation.

Based on CMIP5 model projections, the WNPSH shows a robust signal toward weakening and eastward retreat in the mid troposphere. The weakened WNPSH in the mid troposphere is mainly contributed by weakened westerly wind on the northern flank of WNPSH, which is resulted from weakened meridional temperature gradient and the associated thermal wind. The zonal mean *H* rises fast in the warming climate, which may mislead people on the long term change of WNPSH if the WNPSH is still measured by *H*. We recommend that the WNPSH should be measured in terms of *He* instead of *H*, to better capture the nature of WNPSH in the current warming climate, in particular in climate change studies.

## Methods

### Data and Model

The observational data used in this study include the NCEP/NCAR reanalysis^34^ and the GPCP v2.2 precipitation^35^. Totally 31 coupled models from the CMIP5 are used to construct the MME. The name for the 31 models are listed in Table S1 of Supplementary Information. All the model data are horizontally interpolated onto the 2.5˚ × 2.5˚ grid points before analysis. For each model, only the first realizations for Historical and RCP8.5 runs are adopted. The RCP4.5 run is also adopted in Supplementary Information to corroborate robust forced response across different RCPs. The period of 1950–1999 is used to construct the mean state for observation and Historical run, whereas the period of 2050–2099 is used for the RCP4.5 and RCP8.5 runs (For GPCP data, 1979–1999 period is used due to available data length).

### Inter-model consensus

For scalar field, the inter-model consensus is evaluated by the percentage of models which agree in the sign of projected change with MME. For vector field, i.e., wind, the inter-model consensus for the zonal and the meridional components are calculated separately, and the maximum for these two components is referred to as the inter-model consensus of the vector field.

## Additional Information

**How to cite this article**: He, C. *et al.* Enhanced or Weakened Western North Pacific Subtropical High under Global Warming? *Sci. Rep.* **5**, 16771; doi: 10.1038/srep16771 (2015).

## Supplementary Material

Supplementary Information
