# Supplementary material for: Enhanced or Weakened Western North Pacific Subtropical High under Global Warming?
Source: Sci Rep. 2015 Nov 26;5:16771. doi: 10.1038/srep16771 (PMC4660429; doi:10.1038/srep16771)
Supplement: Supplementary Information [file srep16771-s1.doc]

**Enhanced or Weakened Western North Pacific Subtropical High under Global Warming?**

Chao He^1,2^, Tianjun Zhou2,3[[1]](#footnote-2), Ailan Lin^1^, Bo Wu^2^, Dejun Gu^1^, Chunhui Li^1^ & Bin Zheng^1^

*1 Institute of Tropical and Marine Meteorology (ITMM), Chinese Meteorological Administration (CMA) , Guangzhou, China*

*2 LASG,* *Institute of Atmospheric Physics (IAP), Chinese Academy of Sciences (CAS), Beijing, China*

*3 Joint Center for Global Change Studies (JCGCS),Beijing, China*

**(Supplementary Information)**

**1. Objective metrics on the WNPSH**

The WNPSH is most usually measured by the geopotential height ("*H*") at 500 hPa. However, the global *H* increases as a result of global warming according to the hydrostatic equation^1,2^. Therefore, caution should be taken in discussing the long-term change of WNPSH^2,3^. Although *H* over the WNP rises substantially, stronger increase is seen in the zonal mean *H* within 0˚-40˚N (Fig. S1). Therefore we cannot conclude the WNPSH is enhanced. The systematic increase in *H* should be removed in order to objectively evaluate the intensity and scope of WNPSH.

How to remove the systematic increase component of *H*? There may be three approaches according to previous studies. 1) Remove the regional averaged *H* over 0˚-40˚N, 180˚W-180˚E^2-4^, and the "eddy" component (*He*) is obtained. 2) Remove the zonal mean *H* at each latitude^5^, and the zonal asymmetric component (*Hza*) is obtained. 3) Remove the *H* value at the equator of each longitude^6^, and the meridional asymmetric component (*Hma*) is obtained. Based on the observational record of 1950-1999 period using NCEP/NCAR reanalysis data^7^, we will show evidences that the second approach is not suitable for WNPSH, and the first approach is the best.

Compared with the original *H* field (Fig. S2a), the oval shape of the WNPSH is well captured by the *He* field (Fig. S2b). In both *H* field and *He* field, the centers of the WNPSH (defined as the grid point with a local maximum) are both located at 27.5˚N, 172.5˚E. This superposition is not surprising because the difference between *H* and *He* field is the same constant value at all grids. However, the spatial pattern of the WNPSH is not captured by the *Hza* field (Fig. S2c). The *Hza* field is characterized by a "C"-shaped structure over the North Pacific (the red-colored region over North Pacific in Fig. S2c), rather than the well-known oval shape. The grid points with local maximum *Hza* values are located near the southern tip and the northern tip of the "C"-shaped region, i.e., 15˚N, 175˚W and 40˚N, 152.5˚W.

Why the mean state of WNPSH cannot be captured by *Hza*? This is because the WPNSH is characterized by stronger meridional asymmetry than zonal asymmetry. As shown in Fig. S2a, the meridional *H* gradients on the southern and northern flanks of WNPSH are much stronger than the zonal *H* gradients on its western and eastern flanks. Correspondingly, the winds on the northern and southern flanks of WNPSH are much stronger than those on the western and eastern flanks (See Fig. 1a in the main text). If we subtract the zonal mean *H* at each latitude from the original *H* field, the meridional gradient of *H* will be destroyed. For example, the zonal mean *H* at 27.5˚N is much higher than the zonal means at 15˚N and 40˚N. If the zonal mean *H* at each latitude is subtracted from the original *H* field, a greater value is subtracted from *H* at 27.5N but smaller values are subtracted at 15˚N and 40˚N. Therefore, it is not surprising that the *Hza* at 25˚N is even lower than 15˚N and 40˚N over WNP.

The zonal asymmetric component maintains the zonal gradient of *H* but destroys the meridional gradient, while the meridional asymmetric component maintains the meriidonal gradient of *H* but destroys its zonal gradient. Since the WNPSH is characterized by much stronger meridional gradient than zonal gradient, it can be inferred that the WNPSH can be well captured by *Hma*. This hypothesis is confirmed by Fig. S2d. Compared with Fig. S2a, the spatial pattern of WPNSH is well captured by *Hma* in terms of the shape and the location of WNPSH, and it is also similar to that of the *He* field.

Although the WNPSH can be well measured by *He*, we use *He* instead of *Hma* for the following reasons: 1) *He* is more widely adopted by previous studies^2-4^ than that of *Hma*^6^. In addition, previous study recommended that *He* be used to remove the warming-induced global increase of *H*^2^. 2) *He* is more robust than *Hma*, since the regional averaged value over 0˚-40˚N is less sensitive to noise than the value of a single grid point at the equator.

Stream function (*S*) is also used by previous studies to measure the subtropical high^5,8^. We tested the relationship between *S* and *H*, by comparing the contours with the shading in Fig. S2. It is shown that the *S*-related fields closely follow the *H*-related fields, including the original *S*, eddy *S* (*Se*), zonal asymmetric component (*Sza*) and meridional asymmetric component (*Sma*). Similarly, the *Sza* is not suitable to measure the WNPSH because it cannot capture the mean state of WNPSH, while the *Se* and *Sma* well capture the mean state structure of WNPSH. We have used *Se* to verify the results based on *He* in the main text.

**2. Robust response across different RCPs**

The RCP8.5 is adopted in the main text to investigate the response of WNPSH to anthropogenic greenhouse gases forcing. The RCP8.5 is a high RCP toward a radiative forcing of 8.5 Wm^-2^ by 2100, equivalent to about 1370 ppm CO2 concentration. Here, the results based on RCP4.5 is shown in Figs. S3-S5, to investigate the sensitivity of the forced response to different RCPs. The RCP4.5 is a medium RCP toward 4.5 Wm^-2^ radiative forcing by 2100, equivalent to about 650 ppm CO2 concentration.

Similar as Fig. 2 in the main text, Fig. S3 shows the projected change of *H* and wind at 500 hPa for RCP4.5. Compared with Fig. 2 in the main text for RCP8.5, the increase of *H* is smaller under RCP4.5, which is resulted from smaller increase of temperature. Despite smaller amplitude in forced response, the increase of *H* is agreed by over 75% of the models everywhere over the WNP, and the spatial pattern of projected changes much resembles that of RCP8.5. The increase of *H* on the southern flank of WNPSH is horizontally uniform, associated with little changes of wind. But the increase of *H* on the northern flank of WNPSH is characterized by strong meridional gradient, contrary to the climatological *H* gradient. Corresponding to the weakened *H* gradient, the westerly wind weakens on the northern flank of WNPSH, which is evidenced by easterly wind anomaly agreed by more than 75% of the models (Fig. S3a). The *He*=0 m contour contracts and retreats eastward under RCP4.5, suggesting a weakened WNPSH, which is consistent with RCP8.5 projection elaborated in the main text.

Fig. S4 shows the latitude-height profiles of MME projected changes in zonal wind and temperature. The spatial patterns of RCP4.5 projected changes are similar as RCP8.5 (Fig. 3 in the main text). Although the reduction of westerly wind on the northern flank of WNPSH is weaker than RCP8.5, it is still agreed by over 75% of the models in the mid to upper troposphere (Fig. S4a). The RCP4.5 projected amplitude of warming is generally smaller than RCP8.5, but it is agreed by over 75% of the models at each grid, and is characterized by the same spatial structure as RCP8.5 (Fig. S4b). The weakened meridional temperature gradient on the northern flank of WNPSH is also responsible for the weakened WNPSH under RCP4.5. Similar as in RCP8.5, the inter-model correlation between the reduced meridional temperature gradient and the reduced westerly wind on the northern flank of WNPSH is 0.93 for all the 31 models, and this correlation coefficient is slightly reduced to 0.82 after excluding the two outliers at the lower-left corner (Fig. S5), both exceeding the 99% confidence level.

Under RCP4.5, the model projected changes are consistent with the results shown in the main text under RCP8.5. The results and conclusions in the main text are robust across different RCPs.

**References**

1 Yang, H. & Sun, S. Q. Longitudinal displacement of the subtropical high in the western Pacific in summer and its influence. *Adv Atmos Sci* **20,** 921-933 (2003).

2 Wu, L. & Wang, C. Has the Western Pacific Subtropical High Extended Westward since the Late 1970s? *J Climate* **28,** 5406-5413 (2015).

3 Huang, Y., Wang, H., Fan, K. & Gao, Y. The western Pacific subtropical high after the 1970s: westward or eastward shift? *Clim Dynam* **44,** 2035-2047 (2015).

4 Zhou, T. J. *et al.* Why the Western Pacific Subtropical High Has Extended Westward since the Late 1970s. *J Climate* **22,** 2199-2215 (2009).

5 Enomoto, T., Hoskins, B. J. & Matsuda, Y. The formation mechanism of the Bonin high in August. *Q J Roy Meteor Soc* **129,** 157-178 (2003).

6 Liu, P., Wu, G., Li, W. & Liu, Y. The Three-Dimensional Structure of Subtropical High Belt. *Chinese Journal of Atmospheric Sciences* **24,** 577-584 (2000).

7 Kalnay, E. *et al*. The NCEP/NCAR 40-year reanalysis project. *B Am Meteorol Soc* **77,** 437-471 (1996).

8 Li, W., Li, L., Ting, M. & Liu, Y. Intensification of Northern Hemisphere subtropical highs in a warming climate. *Nat Geosci* **5,** 830-834 (2012).

9 The NCAR Command Language (Version 6.1.2) [Software]. (2013). Boulder, Colorado: UCAR/NCAR/CISL/VETS. http://dx.doi.org/10.5065/D6WD3XH5.

**Table S**1 Information about the 31 models used in this study

| Modeling Group | Model Name | ID |
| --- | --- | --- |
| Commonwealth Scientific and Industrial Research Organization and Bureau of Meteorology (CSIRO-BOM) | ACCESS1.0 | A |
|  | ACCESS1.3 | B |
| Beijing Climate Center, China Meteorological Administration (BCC) | bcc-csm1.1 | C |
| College of Global Change and Earth System Science, Beijing Normal University (GCESS) | BNU-ESM | D |
| Canadian Centre for Climate Modelling and Analysis (CCCMA) | CanESM2 | E |
| National Center for Atmospheric Research (NCAR) | CCSM4 | F |
|  | CESM1-BGC | G |
|  | CESM1-CAM5 | H |
| Centro Euro-Mediterraneo per I Cambiamenti Climatici (CMCC) | CMCC-CM | I |
| Centre National de Recherches Météorologiques / Centre Européen de Recherche et Formation Avancée en Calcul Scientifique (CNRM-CERFACS) | CNRM-CM5 | J |
| Commonwealth Scientific and Industrial Research Organization in collaboration with Queensland Climate Change Centre of Excellence (CSIRO-QCCCE) | CSIRO-Mk3.6.0 | K |
| LASG, Institute of Atmospheric Physics, Chinese Academy of Sciences (LASG-IAP) | FGOALS-g2 | L |
|  | FGOALS-s2 | M |
| NOAA Geophysical Fluid Dynamics Laboratory (NOAA GFDL) | GFDL-CM3 | N |
|  | GFDL-ESM2G | O |
| NASA Goddard Institute for Space Studies (NASA GISS) | GISS-E2-H | P |
|  | GISS-E2-R | Q |
| National Institute of Meteorological Research/Korea Meteorological Administration (NIMR/KMA) | HadGEM2-AO | R |
| Met Office Hadley Centre (additional HadGEM2-ES realizations contributed by Instituto Nacional de Pesquisas Espaciais) (MOHC additional realizations by INPE) | HadGEM2-CC | S |
| Institute for Numerical Mathematics (INM) | inmcm4 | T |
| Institut Pierre-Simon Laplace (IPSL) | IPSL-CM5A-LR | U |
|  | IPSL-CM5A-MR | V |
|  | IPSL-CM5B-LR | W |
| Japan Agency for Marine-Earth Science and Technology, Atmosphere and Ocean Research Institute (The University of Tokyo), and National Institute for Environmental Studies | MIROC-ESM | X |
|  | MIROC-ESM-CHEM | Y |
| Atmosphere and Ocean Research Institute (The University of Tokyo), National Institute for Environmental Studies, and Japan Agency for Marine-Earth Science and Technology | MIROC5 | Z |
| Max-Planck-Institut für Meteorologie (Max Planck Institute for Meteorology) (MPI-M) | MPI-ESM-LR | a |
|  | MPI-ESM-MR | b |
| Meteorological Research Institute (MRI) | MRI-CGCM3 | c |
| Norwegian Climate Centre (NCC) | NorESM1-M | d |
|  | NorESM1-ME | e |

**Fig. S1 Projected changes in zonal mean *H* and regional mean *H* over WNP.** **(a)** is for RCP4.5 and **(b)** is for RCP8.5. The wide yellow bar shows the increase of *H* for the zonal mean (0˚-40˚N, 180˚W-180˚E), and the thin black bar shows the increase of *H* for the regional mean over WNP (10˚-30˚N, 120˚-180˚E). The leftmost bar represent the MME, and other bars represent the individual models. Please refer to Supplementary Table S1 for which model each letter stands for. This plot was created by NCAR Command Language^9^.

**Fig. S2 The mean state of WNPSH at 500 hPa in observation**, as revealed by geopotential height (shading, unit: m) and stream function (contours, unit: m^2^s^-1^). **(a)** The original geopotential height and stream function. **(b)** The eddy components obtained by subtracting the regional average over 0˚-40˚N, 180˚W-180˚E. **(c)** The zonal asymmetric components obtained by subtracting the zonal mean at each latitude. **(d)** The meridional asymmetric components obtained by subtracting the value of the equator at each longitude. The contour interval for stream function related fields is 3×10^6^ m^2^s^-1^, with the negative contours dashed. The stars indicate the locations with local maxima. This plot was created by NCAR Command Language^9^.

**Fig. S3 Same as Fig. 2 in the main text but for RCP4.5.** The increase of *H* in (a) is agreed by over 75% of the individual models everywhere, and it is not particularly marked. This plot was created by NCAR Command Language^9^.

**Fig. S4 Same as Fig. 3 in the main text but for RCP4.5.** The white crossed region indicates the inter-model consensus is above 75%. The increase of temperature in (b) has exceeded 75% inter-model consensus everywhere, and the white-crosses are omitted. This plot was created by NCAR Command Language^9^.

**Fig. S5** **Same as Fig. 4 in the main text but for RCP4.5**. The inter-model correlation coefficient for all the 31 models is marked on the upper-right corner of the plot, and the correlation coefficient after excluding the two outliers at the lower-left corner of the plot is marked within the parenthesis. This plot was created by NCAR Command Language^9^.

1. Corresponding author address: Tianjun Zhou, P.O. Box 9804, Institute of Atmospheric Physics (IAP), Beijing, China

   Email: zhoutj@lasg.iap.ac.cn [↑](#footnote-ref-2)
